# Transmission estimation at the quantum Cramér-Rao bound with macroscopic quantum light

**DOI:** 10.1140/epjqt/s40507-022-00154-x

**Published:** 2022-12-22

**Authors:** Timothy S. Woodworth, Carla Hermann-Avigliano, Kam Wai Clifford Chan, Alberto M. Marino

**Affiliations:** 1 Homer L. Dodge Department of Physics and Astronomy, The University of Oklahoma, Norman, Oklahoma 73019 USA; 2 Center for Quantum Research and Technology, The University of Oklahoma, Norman, Oklahoma 73019 USA; 3 Departamento de Física, Facultad de Ciencias Físicas y Matemáticas, Universidad de Chile, Santiago, Chile; 4 ANID – Millennium Science Initiative Program – Millennium Institute for Research in Optics (MIRO), Santiago, Chile; 5 OAM Photonics LLC, San Diego, California 92126 USA; 6 Quantum Information Science Section, Oak Ridge National Laboratory, Oak Ridge, Tennessee 37381 USA; 7 Quantum Science Center, Oak Ridge National Laboratory, Oak Ridge, Tennessee 37381 USA

**Keywords:** Quantum metrology, Quantum sensing, Quantum Cramér-Rao bound, Quantum Fisher information, Quantum optics, Transmission estimation

## Abstract

The field of quantum metrology seeks to apply quantum techniques and/or resources to classical sensing approaches with the goal of enhancing the precision in the estimation of a parameter beyond what can be achieved with classical resources. Theoretically, the fundamental minimum uncertainty in the estimation of a parameter for a given probing state is bounded by the quantum Cramér-Rao bound. From a practical perspective, it is necessary to find physical measurements that can saturate this fundamental limit and to show experimentally that it is possible to perform measurements with the required precision to do so. Here we perform experiments that saturate the quantum Cramér-Rao bound for transmission estimation over a wide range of transmissions when probing the system under study with a continuous wave bright two-mode squeezed state. To properly take into account the imperfections in the generation of the quantum state, we extend our previous theoretical results to incorporate the measured properties of the generated quantum state. For our largest transmission level of 84%, we show a 62% reduction over the optimal classical protocol in the variance in transmission estimation when probing with a bright two-mode squeezed state with −8 dB of intensity-difference squeezing. Given that transmission estimation is an integral part of many sensing protocols, such as plasmonic sensing, spectroscopy, calibration of the quantum efficiency of detectors, etc., the results presented promise to have a significant impact on a number of applications in various fields of research.

## Introduction

The second quantum revolution seeks to develop new technology that can take advantage of quantum resources and lead to practical applications of quantum mechanics. These include, for example, quantum computing [1] to solve problems intractable for classical computers, such as factorization [2, 3] and database searching [4]; quantum cryptography for the transfer of information with absolute security [5–7]; quantum imaging for enhanced resolution [8–10] for applications such as imaging of biological samples without damage [11, 12]; and quantum metrology [13, 14] for enhanced measurements. In particular, quantum metrology, which is the focus of this paper, seeks to use quantum resources, such as quantum states of light, to enhance systems and measurements beyond what is possible with classical resources. Such quantum enhancements have already been demonstrated in real-life devices, such as in the advanced Laser Interferometer Gravitational-Wave Observatory (LIGO) experiments [15] in which the use of squeezed light has enhanced the ability to detect gravitational waves.

The precision with which a parameter can be estimated is given by the inverse of the uncertainty in the estimation of its value. To understand the limits in the precision that can be achieved through the use of quantum resources, it is therefore necessary to know the fundamental minimum uncertainty in the estimation of the parameter of interest. For a given state and system under study, the minimum variance of the mean of the parameter to estimate is bounded from below by the quantum Cramér-Rao bound (QCRB) [14, 16–18]. Despite the quantum descriptor of this bound, it is not limited to quantum states but is derived from the quantum representation of the classical or quantum state used to probe the system under study and the use of quantum techniques to optimize over all possible measurements. Thus, the QCRB is independent of the measurement performed on the probing state after it interacts with the system and depends only on the response of the system to the parameter of interest and the state probing the system. As a result, if a given measurement uncertainty saturates the QCRB, then that measurement is the optimal one and no other measurement can provide a further reduction in uncertainty. Furthermore, the ratio between the QCRBs when probing with a quantum state and with the optimal classical state establishes the maximum quantum enhancement that can be achieved. Therefore, measuring a parameter at the QCRB with a quantum state ensures the maximum precision and quantum enhancement for that particular state.

Here we focus on the estimation of transmission, which is the basis of many sensing applications that have benefitted from the use of quantum states. For example, quantum states of light have enhanced plasmonic sensors [19], two-photon absorption spectroscopy [20–22], and the calibration of the quantum efficiency of detectors [23–31]. For transmission estimation, it has been shown theoretically that single-mode states with reduced intensity noise [32, 33] and two-mode states with reduced intensity-difference noise [34, 35] can provide a quantum-based enhancement. Specifically, it is known that the Fock state [33] and the vacuum two-mode squeezed state (vTMSS) [34] have the lowest possible QCRB for transmission estimation, referred to here as the ultimate bound. However, these states can only be generated at very low power levels [36–40]. Since the QCRB for transmission estimation scales inversely with the number of photons [33–35], the low photon numbers of these states limits the absolute uncertainty in transmission estimation that can be achieved. As a result, these states are, in general, not able to surpass the corresponding classical state-of-the-art and their applicability to real-life sensing applications is limited. To overcome this limitation, it is possible to use bright quantum states of light, *i.e.* states with a mean field amplitude much larger than their quadrature fluctuations, that can be generated with orders magnitude larger number of photons. While such states are not able to reach the ultimate bound in general, they can achieve a lower overall QCRB and surpass the classical state-of-the-art [19]. Here we specialize to the use of the bright two-mode squeezed state (bTMSS), as it approaches the ultimate bound at high levels of squeezing [35] and can be generated at high powers [41–43]. Thus, in practice, the bTMSS gives a better absolute sensitivity in the estimation of transmission than the Fock or vTMSS given that it is a macroscopic quantum state. In addition, we have previously identified a measurement that can in theory saturate the QCRB and can be implemented with current technology [35].

The importance of quantum enhanced transmission estimation has led to recent experimental works that demonstrated that a quantum advantage is possible [44–50]. As opposed to previous experiments, we show for the first time that it is possible to perform measurements that saturate the QCRB for transmission estimation over a broad range of transmission levels (limited only by our accessible transmission through the system) and do so without any free parameters. Furthermore, the use of bright quantum states of light offers significant advantages in the absolute sensitivity that can be achieved, as described above. To our knowledge, the only other experiment that has been able to saturate the QCRB for transmission estimation was performed with single photons in 2017 by Whittaker *et. al* [46]. They saturated the QCRB for Fock states using a heralded single photon source, though only over a very limited transmission range from 10% to 30% where the quantum advantage is small. That same year, Sabines-Chesterking *et. al* [45] attempted to reach the QCRB for Fock states with active feed-forwarding of single photons to avoid the probabilistic operation due to postselection. However, due to technical limitations they were not able to saturate the bound. The bright nature of the quantum states used in our experiment results in absolute sensitivities many orders of magnitude larger than for either of these experiments, as needed to surpass the classical state-of-the-art.

Experiments done with squeezed light, on the other hand, have not been able to experimentally verify or even attempted to saturate the QCRB. For example, for the case of a single-mode squeezed state, recent work by Atkinson *et. al* [49] was only assumed to saturate the QCRB given measured trends. An absolute comparison between the measured uncertainty and the theoretical absolute uncertainty given by the QCRB was not performed. Furthermore, their work was limited to the measure of a transmission modulation peak height at a single transmission and studied the degree of quantum advantage for different squeezing levels and detection bandwidths. Earlier work by D’Auria *et. al* [44] obtained a quantum-based enhacement; however, an analysis of the QCRB was not performed and the level of enhacement did not approach this bound. Finally, the work done with two-mode squeezed states has focused on obtaining a quantum advantage and not on saturating the QCRB. In 2017, Moreau *et. al* [47] showed that the vTMSS does lead to reduced uncertainty in transmission estimation compared to a coherent state, but their measurement was only able to achieve this for transmissions above 30%. Nearly concurrently, Losero *et. al* were able to obtain a larger quantum enhancement due to a larger level of squeezing [48]. Then, in 2021 Li *et. al* [50] showed a quantum advantage with bTMSS for transmissions above 40%. While all these experiments obtained a quantum advantage, none of them were able to saturate the QCRB.

## Experimental setup and procedure

**Figure 1:**
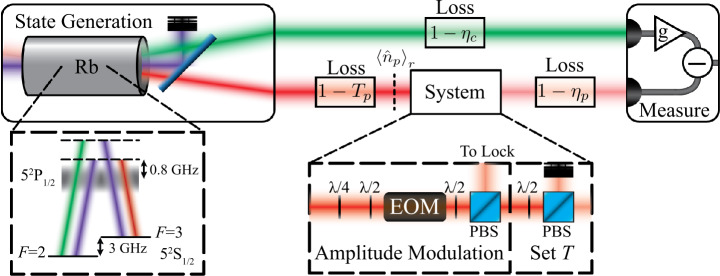
Experimental setup for transmission estimation at the QCRB with a bTMSS. A continuous wave bTMSS is generated in a ^85^Rb vapor cell via a FWM process in a double-Λ configuration in the D1 line, as shown in the “state generation” inset. The response of the atomic medium leads to a bTMSS from the FWM process with a bandwidth of the order of 10 MHz [42]. To generate the bTMSS, a strong pump beam (shown in purple) is combined with a weak probe beam (shown in red) to generated quantum correlated probe and conjugate (shown in green). The probe beam is used to probe the system under study, while the conjugate beam serves as the reference for the transmission estimation. We consider losses in the probe mode both before and after the system under study and losses in the conjugate mode. The “system” inset shows the configuration that is used to emulate a transmissive system. An electro-optic modulator (EOM) is used in an amplitude modulation configuration with the reflection from the polarizing beam splitter (PBS) after the EOM used to stabilize it (see Appendix A.1). After the amplitude modulation section of the system, a half waveplate and PBS are used to control the mean transmission through the system. The transmission of the system is given by the mean transmission of the EOM setup and the Set *T* half waveplate and PBS. An optimal intensity-difference measurement of the probe and conjugate modes, with electronic attenuation of the photocurrent of the detected conjugate mode, is used to obtain the uncertainty in the estimation of the transmission, $\langle \Delta ^{2}T \rangle $.

For transmission estimation with a bTMSS, one mode is used to probe the system under study, and is therefore called the probe mode, while the other mode is used as a reference. For this study, we consider the number of photons in the probe mode that interact with the system, $\langle \hat{n}_{p} \rangle _{r}$ in Fig. 1, as the resources for the parameter estimation. Many systems have a limit in the number of photons they can interact with without damage or other adverse effects and this is the typical limiting factor for parameter estimation. It is for such systems that quantum states, which have a lower QCRB than classical states for the same number of probing photons, can provide an enhancement in precision that can surpass the classical state-of-the-art for practical applications.

The configuration that we use is shown in Fig. 1. We generate a continuous wave bTMSS with a four-wave mixing (FWM) process, which is based on a double-Λ configuration, as shown in the “state generation” inset in Fig. 1. In this non-linear process, two photons from a strong pump beam are absorbed to simultaneously create one photon in the probe and one in a new beam commonly referred to as the conjugate, which serves as the reference for estimating the transmission. If the probe and conjugate modes are not seeded (input vacuum sates) then a vTMSS is generated. However, if either mode is seeded, typically with a coherent state, the generation rate of photons is increased. If the power of the seeding mode(s) is large enough that the generation rate of stimulated photons is much larger than the rate for spontaneously generated photons, then the state is a bTMSS.

We implement the FWM process in the D1 line of ^85^Rb in a 12 mm long hot vapor cell heated to 120°C. A strong pump (600 mW of power and $1/e^{2}$ radius waist of 700 *μ*m) is combined with the seeding probe mode (7 *μ*W of power and $1/e^{2}$ radius waist of 400 *μ*m) at an angle of 0.4° at the center of the Rb cell. The pump beam is generated with a Ti:Sapph laser at 795 nm while the seeding beam is generated by taking a portion of the pump and downshifting its frequency by 3.04 GHz via double passing an acousto-optic modulator (AOM). Before seeding the FWM process, a cleanup cavity (Newport SuperCavity model SR-140-C) is used to filter out any technical noise in the probe mode, such that it is shot noise limited at 1.5 MHz. For these parameters, the FWM has a gain of 11.4 and the generated probe and conjugate have a measured intensity-difference noise 8.0 dB below the shot noise, after subtracting the electronic noise. To keep the number of photons probing the system, $\langle \hat{n}_{p} \rangle _{r}$, constant throughout the experiment, we lock the probe seed power before the Rb vapor cell and stabilize the gain of the FWM by locking the temperature of the cell, the pump power, and the frequency of the laser. The frequency of the laser, and therefore of the pump and probe, is locked via the conjugate power, as explained in Appendix A.1.

To emulate a system with linear transmission, we use the configuration shown in the “system” inset of Fig. 1, which consists of two parts. The first part modulates the transmission, as needed to determine the transmission modulation at which the signal-to-noise ration (SNR) is equal to one and thus the uncertainty in transmission estimation (see Appendix A.3). The second part sets the mean transmission through the system, *T*. To modulate the transmission we use an electro-optic modulator (EOM) in an amplitude modulation configuration. For light incident on the EOM with a polarization that is not aligned to one of its axes, the EOM introduces a phase shift between the field components along the directions of the EOM crystal axes. This leads to a change in the polarization of the light that can be controlled by a voltage applied across the EOM crystal. When the EOM is followed by a half waveplate and a polarizing beam splitter (PBS), the polarization modulation is converted into a transmission modulation. A quarter waveplate and a half waveplate before the EOM give complete control over the polarization of the incident light, thus allowing for control of the transmission modulation properties such as the transmission modulation amplitude *δT* (define as the standard deviation of the modulation) introduced by the system. For the second part of the system, we use another half waveplate and PBS to explore the QCRB dependence on transmission.

Finally, an optimized intensity-difference measurement is performed on the optical state. As we have previously shown, in theory this measure saturates the QCRB for transmission estimation with a bTMSS [35]. This measurement is similar to a balanced intensity-difference measurement, where the measured photocurrents of the two modes are subtracted, except for an electronic attenuation of the photocurrent of one of the modes being performed before the subtraction. In our experiment, the photocurrent of the detected conjugate mode is electronically attenuated to maximize the cancellation of the intensity noise of the detected probe mode.

**Figure 2:**
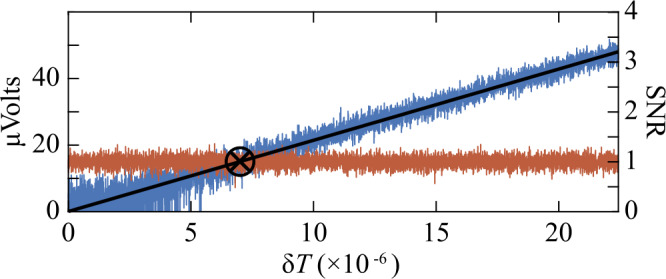
Measured signal and noise for *T*=15%, as a function of the transmission modulation amplitude. The signal trace (blue), obtained while ramping the modulation amplitude, and the optimized intensity-difference noise trace (red), obtained with the modulation off, measured with a spectrum analyzer are shown in volts on the left *y*-axis after subtraction of the electronic noise. The right *y*-axis shows the corresponding SNR obtained by taking the ratio of the signal to the mean value of the optimized intensity-difference noise. The value on the *x*-axis at which the signal is equal to the noise (or SNR = 1), marked by a circled “X”, gives the transmission modulation amplitude, $\delta _{T}$, that can then be used to obtain the uncertainty in the estimation of transmission, $\langle \Delta ^{2}T \rangle $, for the bTMSS (see Appendix A.3).

To determine the uncertainty in the estimation of the transmission, $\langle \Delta ^{2}T \rangle $, we use a spectrum analyzer to determine the point at which a calibrated (see Appendix A.2) transmission modulation amplitude *δT* results in a measured SNR = 1. As shown in Appendix A.3, at this point the square of *δT* is equal to the uncertainty in the estimation of transmission. To find the value of *δT* at which SNR = 1, we ramp down the transmission modulation amplitude introduced with the EOM to determine the value at which it is equal to the noise, as indicated by the circled “X” mark in Fig. 2.

We start with a large fixed modulation amplitude for 2 seconds, not shown in Fig. 2, to reduce the effects of ringing when the ramping cycle is repeated, followed by a linear reduction of the modulation amplitude to zero over 14 seconds. To obtain repeatable measurements, the EOM transmission modulation amplitude is locked (as described in Appendix A.1) during the entire procedure and the ramping is done by changing the setpoint of the lock, such that the EOM modulation voltage follows to produce a linear ramp of the transmission modulation amplitude. We measure the output of the optimized intensity-difference measurement with a spectrum analyzer in volts with the ramp and without a modulation. The signal, shown in blue in Fig. 2 (volts scale on the left), is obtain by taking the measured trace with the ramp on and subtracting, in quadrature, the mean optimized intensity-difference noise, which is given by the measured trace without a modulation (red trace in Fig. 2).

The SNR, given on the right scale in Fig. 2, is obtained by the ratio, in volts, of the signal to the mean optimized intensity-difference noise. The obtained SNR is then fitted with a line (black solid line in Fig. 2) to find the value of *δT* at which the $\mathrm{SNR} =1$ such that $\langle \Delta ^{2}T \rangle =(\delta T|_{\mathrm{SNR} = 1})^{2}$ for our measurement, as shown in Appendix A.3. This process is repeated for different transmissions *T*, set with the second part of the “system” defined in Fig. 1, to determine $\langle \Delta ^{2}T \rangle $ for our measurement configuration across a wide transmission range. During this process, the transmission is modulated with the EOM at 1.5 MHz given that at this frequency the probe seed beam for the FWM is shot noise limited after passing through the cleanup cavity. This ensures that the measurements are not contaminated with technical noise and are thus dominated by the quantum statistics of the probing light, as needed to perform measurements at the QCRB.

We take a total of 20 sets of transmission uncertainty measurements. For each set we start at the maximum possible mean transmission, $\approx 85\%$, and lower it in steps of 5% to a minimum mean transmission of 10%. At each transmission level we take one trace with the transmission modulation ramp on and one with the modulation off in order to calculate the SNR. We take one complete series of 16 mean transmissions and then return to the maximum transmission to take the next set. This approach allows us to rule out systematic effects, such as changes in the level of squeezing or probing power, that could also lead to changes in the measured uncertainties. Additionally, each mean transmission is measured for every set by first measuring the intensity of the probe mode before the system under study, thus reducing any biasing of the transmission due to power drifts.

As has been previously shown, the QCRB for transmission estimation scales inversely with the number of probing photons [33–35]. Thus, in order to perform a direct comparison between the measured transmission uncertainties and the QCRB without any free parameters, a proper calibration of the number of photons used to probe the system under study is essential. We perform this calibration by measuring the photon flux (which is proportional to the probe optical power, set to 80 *μ*W in the experiment) and multiplying it by the effective measurement time, *t*, for our setup, which is determined by the resolution bandwidth (RBW) of the spectrum analyzer. As outlined in Appendix A.4, the effective measurement time for our spectrum analyzer is ≈0.44/RBW, which leads to $t=8.63 \mu $s for the RBW of 51 kHz used in the experiments. This gives a mean number of probing photons of $\langle \hat{n}_{p} \rangle _{r} \sim 10^{9}$.

## Results

**Figure 3:**
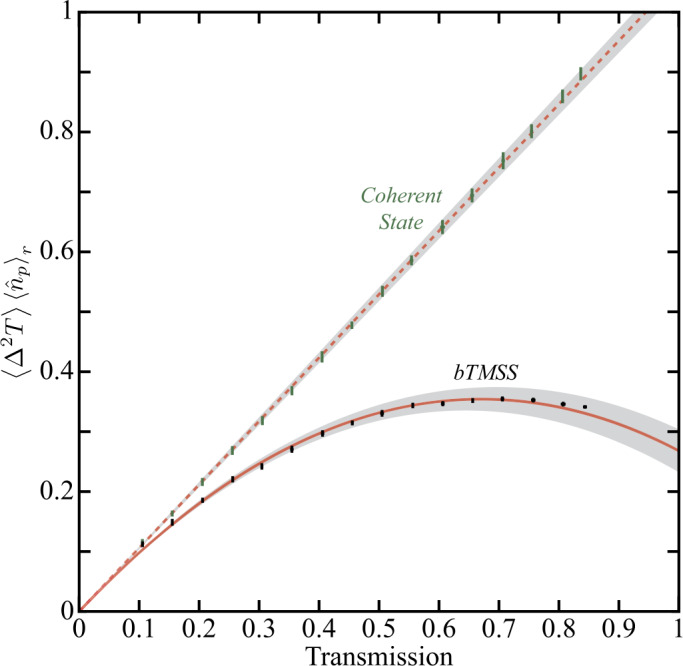
Measured uncertainties in transmission estimation as a function of mean transmission *T*. The results are plotted as the product $\langle \Delta ^{2}T \rangle \langle \hat{n}_{p} \rangle _{r}$ to make them independent of probe power or detection bandwidth used for the estimation and obtain a better comparison with the QCRB. Black and green data points correspond to the bTMSS and coherent state measurements, respectively. The vertical lines, not always visible, around each data point are the one sigma standard deviation over the 20 measurements performed at each mean transmission. The red lines are the QCRB predictions for the generated bTMSS (solid) with estimated squeezing parameter $s=2.04\pm 0.02$ and internal source probe losses of $T_{a}=71\%\pm 2\%$ (see Appendix A.5) and coherent state (dashed), with the shaded grey regions giving the one sigma uncertainty in the theoretical predictions to account for uncertainties in the required calibrations of $T_{p}=97.3\%\pm 1\%$, $\eta _{p}=94.5\%\pm 2\%$, and $\eta _{p}=91.9\%\pm 2\%$. No free parameters or fittings were used for the QCRB plots, which take into account all the experimental imperfections that were independently calibrated. As can be seen, our measurements with bTMSS and coherent states saturate their respective QCRB.

The results for the bTMSS are shown as black data points (black dots with one sigma error bars) in Fig. 3. In order to compare our measured transmission uncertainties with the QCRB in a way that is independent of the probing power or detection bandwidth, we plot the product of the transmission estimation variance and mean number of probing photons, $\langle \Delta ^{2}T \rangle \langle \hat{n}_{p} \rangle _{r}$, which is independent of the number of photons (resources) used to probe the system, as a function of the mean transmission *T*. As an additional check to our procedure and to obtain a measure of the degree of quantum enhancement possible with the bTMSS, we repeat the experiment with the optimal classical configuration using a coherent state. To do so, we remove the Rb vapor cell but keep everything else the same between the measurements with a coherent state and a bTMSS. Since there is only one mode for the coherent state, the optimized intensity-difference measurement simplifies to an intensity measurement of the coherent state. The intensity measurement has also been shown to saturate the QCRB for transmission estimation with a coherent state [35]; thus, we are comparing our measurements with the bTMSS to the best possible classical transmission estimation. The results obtained with the optimal classical configuration are shown as green data points (green dots with one sigma error bars) in Fig. 3. As can be seen, for our maximum transmission of 84% we obtained a quantum advantage over the optimal classical configuration by a factor of 2.6 through the use of a bTMSS with −8.0 dB of balanced intensity-difference squeezing. This corresponds to a reduction of 62% in the variance in the estimation of transmission. The error bars on the data points for both the bTMSS and the coherent state correspond to a one sigma standard deviation over the 20 measurements performed at each mean transmission for both the mean transmission and the uncertainty in the estimation of transmission.

To compare the measured transmission uncertainties with the corresponding QCRB, one needs to properly take into account the quantum state that is used to probe the system under study. We previously showed that the QCRB for transmission estimation using a pure bTMSS, with losses in the probe mode before and after the system and loss in the conjugate mode (as shown in Fig. 1), is given by [35]

1 $$ \bigl\langle \Delta ^{2}T \bigr\rangle _{\text{bTMSS}}\ge \frac{T}{\eta _{p} \langle \hat{n}_{p} \rangle _{r}}- \frac{T^{2}}{ \langle \hat{n}_{p} \rangle _{r}}T_{p}H_{c} \bigl[1-\operatorname{sech}(2s) \bigr], $$

where $\langle \hat{n}_{p} \rangle _{r}$ is the number of photons in the probe mode incident on the system, $T_{p}$ and $\eta _{p}$ are the transmissions before and after the system, respectively, and *s* is the squeezing parameter that controls the rate of generation of photon pairs, *i.e.* the FWM gain, which also sets the quantum correlations between the probe and conjugate modes [41, 51, 52]. Additionally

2 $$ H_{c}= \frac{ (2\eta _{c}-1 ) \left[1+2\sinh ^{2}(s) \right]}{1+2\eta _{c}\sinh ^{2}(s)}, $$

where $\eta _{c}$ is the transmission of the conjugate mode. It should nevertheless be pointed out that the assumptions leading to Eq. (1) are not exactly valid for our experimental implementation, as the generated state is not a pure bTMSS. This is due to internal losses in the atomic medium used to implement the FWM and the fact that the two-mode squeezing operator does not commute with the loss operator, which means that we cannot consider the source as a perfect squeezer followed by the losses introduced by the atomic system.

To have a more accurate characterization of the generated quantum state, and thus correctly set the QCRB, we consider a model for the source that consists of an infinite series of alternating infinitesimal layers of two-mode squeezers and beam splitters (to model internal loss) [41, 53]. This allows us to obtain a full description of the generated state in terms of the covariance matrix, with matrix elements $\sigma _{ij}=\{\hat{A}_{i}-d_{i},\hat{A}_{j}^{\dagger}-d_{j}^{*}\}$, and displacement vector, $\vec{d}=\langle \vec{\hat{A}}\rangle $, where $\vec{\hat{A}}={\left(\begin{array}{cccc} {\hat{a}}_{p} & {\hat{a}}_{c} & {\hat{a}}_{p}^{†} & {\hat{a}}_{c}^{†} \end{array}\right)}^{T}$ is a field operator vector and $\{\cdot ,\cdot \}$ is the anti-commutation relation. With this model and the assumption that there is no absorption of the conjugate mode due to the atomic medium, given that its frequency is far-off resonance from any transition, the displacement vector and covariance matrix for the generated bTMSS take the form

3 $$\vec{d}=\alpha \left(\begin{array}{c} \sqrt{T_{p}T\eta_{p}\sqrt{T_{a}}}\left[\cosh\left(\frac{\xi }{4}\right)+\frac{\ln(T_{a})\sinh\left(\frac{\xi }{4}\right)}{\xi }\right]\\ \frac{4sT_{a}^{\frac{1}{4}}\sinh\left(\frac{\xi }{4}\right)}{\xi }\\ \sqrt{T_{p}T\eta_{p}\sqrt{T_{a}}}\left[\cosh\left(\frac{\xi }{4}\right)+\frac{\ln(T_{x})\sinh\left(\frac{\xi }{4}\right)}{\xi }\right]\\ \frac{4sT_{a}^{\frac{1}{4}}\sinh\left(\frac{\xi }{4}\right)}{\xi } \end{array}\right),$$

4 $$\sigma =\left(\begin{array}{cccc} T_{p}T\eta_{p}\sigma_{pp}+1-T_{p}T\eta_{p} & 0 & 0 & \sqrt{T_{p}T\eta_{p}\eta_{c}}\sigma_{pc}\\ 0 & \eta_{c}\sigma_{cc}+1-\eta_{c} & \sqrt{T_{p}T\eta_{p}\eta_{c}}\sigma_{pc} & 0\\ 0 & \sqrt{T_{p}T\eta_{p}\eta_{c}}\sigma_{pc} & T_{p}T\eta_{p}\sigma_{pp}+1-T_{p}T\eta_{p} & 0\\ \sqrt{T_{p}T\eta_{p}\eta_{c}}\sigma_{pc} & 0 & 0 & \eta_{c}\sigma_{cc}+1-\eta_{c} \end{array}\right),$$

where

5 $$\begin{aligned}& \sigma _{pp} = 1+ \frac{32s^{2}\sqrt{T_{a}}\sinh ^{2}\left(\frac{\xi}{4}\right)}{\xi ^{2}}, \end{aligned}$$

6 $$\begin{aligned}& \sigma _{pc} = \frac{4s\sqrt{T_{a}} \left[\xi \sinh \left(\frac{\xi}{2}\right)-2\ln (T_{a})\sinh ^{2}\left(\frac{\xi}{4}\right) \right]}{\xi ^{2}}, \end{aligned}$$

7 $$\begin{aligned}& \sigma _{cc} = \sqrt{T_{a}} - 1+ \frac{\sqrt{T_{a}} \left\{\cosh \left(\frac{\xi}{2}\right) \left[\xi ^{2}+\ln ^{2}(T_{a}) \right] - \ln (T_{a}) \left[\ln (T_{a})+2\xi \sinh \left(\frac{\xi}{2}\right) \right] \right\}}{\xi ^{2}}, \end{aligned}$$

$T_{\text{a}}$ is the product of the transmissions of all beam splitters in the model for the probe mode, *s* is the sum of all squeezing parameters of the squeezers in the model, and $\xi =\sqrt{16s^{2}+\ln ^{2}(T_{\text{a}})}$.

The QCRB can then be calculated following the method for Gaussian states given by Šafránek [54], where the uncertainty in the estimation of transmission satisfies

8 $$ \bigl\langle \Delta ^{2}T \bigr\rangle \ge \biggl(2 \frac{\partial \vec{d}^{\dagger}}{\partial T} \sigma ^{-1} \frac{\partial \vec{d}}{\partial T} \biggr)^{-1} $$

in the bright limit obtained when the stimulated photon pair generation dominates over the spontaneous pair generation [35]. For the generated state, we find that the QCRB takes the form

9 $$ \bigl\langle \Delta ^{2}T \bigr\rangle _{\text{bTMSS}}\ge \frac{T}{\eta _{p} \langle \hat{n}_{p} \rangle _{r}}- \frac{T^{2}}{ \langle \hat{n}_{p} \rangle _{r}}T_{p}H'_{c} \frac{32s^{2}\sqrt{T_{\text{a}}}\sinh ^{2} \left(\frac{\xi}{4} \right)}{ \xi ^{2}(\sqrt{T_{\text{a}}}-1)+\Gamma}, $$

where

10 $$ \Gamma =\sqrt{T_{\text{a}}} \biggl\{ \cosh \biggl(\frac{\xi}{2} \biggr) \bigl[\xi ^{2}+\ln ^{2}(T_{\text{a}}) \bigr]-\ln (T_{\text{a}}) \biggl[ \ln (T_{\text{a}})+2\xi \sinh \biggl( \frac{\xi}{2} \biggr) \biggr] \biggr\} $$

and

11 $$ H'_{c}=\frac{2\eta _{c}-1}{\eta _{c}} \biggl\{ 1+ \frac{\xi ^{2}(\eta _{c}-1)}{\xi ^{2}[1+\eta _{c}(\sqrt{T_{\text{a}}}-2)]+\eta _{c}\Gamma} \biggr\} . $$

The factor $H'_{c}$ plays the same role as $H_{c}$ in Eq. (1), such that $H'_{c}=1$ when $\eta _{c}=1$, $H'_{c}=0$ when $\eta _{c}=1/2$, and $H'_{c}<0$ when $\eta _{c}<1/2$. The QCRB for the coherent state can then be obtained by setting $s=0$ in either Eq. (1) or (9), to give

12 $$ \bigl\langle \Delta ^{2}T \bigr\rangle _{\text{coh}}\ge \frac{T}{\eta _{p} \langle \hat{n}_{p} \rangle _{r}}, $$

which scales linearly with transmission.

To evaluate the QCRB for our system, we need to consider the losses external to the system that do not form part of the measured transmission uncertainties shown in Fig. 3, but increase the QCRB, as can be seen from Eqs. (1) and (9). Imperfect probe transmission before the system, $T_{\text{p}}$, comes from the Rb vapor cell output window, polarization filter used to separate the pump mode from the probe and conjugate modes, and the various mirrors and lenses used to propagate the probe mode to the system under study. The transmission through the cell window was measured to be $98.8\%\pm 1$% and the propagation transmission between the system and Rb vapor cell, after the cell window, was measured to be $98.4\%\pm 1$% for a total transmission before the system, $T_{\text{p}}$, of $97.3\%\pm 1\%$. Transmission in the path of the probe after the system, $\eta _{p}$, comes mainly from the photodiode’s quantum efficiency, which we approximate from the data sheet and previous measurements to be of $94.5\%\pm 2$% [41]. The conjugate mode transmission, $\eta _{c}$, is equal to the combination of the probe transmission both before and after the system under study, $\eta _{c}=T_{p}\eta _{p}$, as the probe and conjugate modes share many optical elements and the quantum efficiencies of the photodiodes are the same for the probe and conjugate. This leads to a total conjugate mode transmission, $\eta _{c}$, of $91.9\%\pm 2\%$. We also need to estimate the effective squeezing parameter *s* and probe loss due to atomic absorption $T_{a}$. We do so by measuring the balanced intensity-difference noise and the single beam intensity noises of the probe and conjugate modes by going around the system under study. We then compare these values, after backtracking the propagation and detection losses ($T_{p}$, $\eta _{p}$, and $\eta _{c}$), with the corresponding values obtained from the model of the source composed of layers of squeezers and losses to find the parameters of the source (see Appendix A.5 for the optimization procedure). Following this procedure we find values of $s=2.04\pm 0.02$ and $T_{a}=71\%\pm 2\%$.

The solid and dashed red lines in Fig. 3 correspond to the QCRB predictions given by Eqs. (9) and (12) for the bTMSS and coherent state, respectively, after taking into account all the experimental imperfections. The shaded regions around these lines represent the theoretical one sigma uncertainty in the calculated QCRB due to uncertainties in the estimation of the losses and the parameters of the generated bTMSS. As can be seen, the measured data is well within the predicted QCRB for both the bTMSS and coherent state, which shows that the measurements performed saturate the QCRB for transmission estimation over the accessible transmission range without any free parameters in the theory. This indicates that the transmission estimation measurements performed are optimal and no further enhancements are possible with the optical states that are used.

One of the reasons we consider the use of a bTMSS is that it approaches the ultimate bound in transmission estimation as the level of squeezing increases [35], even for the imperfect bTMSS generated by our FWM source. This can be seen if one takes the limit of infinite squeezing, $s\rightarrow \infty $, and perfect conjugate detection, in Eq. (1) or (9). In this limit the equations reduce to

13 $$ \bigl\langle \Delta ^{2}T \bigr\rangle _{\text{ult}}\ge \frac{T}{\eta _{p} \langle \hat{n}_{p} \rangle _{r}}- \frac{T^{2}}{ \langle \hat{n}_{p} \rangle _{r}}T_{p}, $$

which corresponds to the ultimate bound in transmission estimation [35]. While we are not at the ultimate bound, the QCRB for the bTMSS does approach it. For example, at our maximum transmission of 84%, where we obtain the maximum quantum enhancement in transmission estimation, we are only a factor of around 1.7 away from the ultimate bound. Furthermore, if we make a conservative comparison of our results with a bTMSS to the ultimate bound with the largest possible Fock state that has been theoretically proposed as viable with current technology [40], our absolute precision would be ∼10^7^ times larger due to the large number of probing photons, $\langle \hat{n}_{p} \rangle _{r}$, in our bTMSS.

## Summary

We performed transmission estimation measurements that saturate the QCRB for both a bTMSS and a coherent state with easily accessible measurement techniques. This fundamental limit was saturated across a broad transmission range of 84% to 10%. We also showed a 62% reduction in the variance in transmission estimation with respect to the optimal classical configuration at 84% transmission when using a bTMSS with −8.0 dB of balanced intensity-difference squeezing. For the number of photons used in our experiment, the absolute uncertainty in transmission for a mean transmission of 84% was $1.11\pm 0.01\times 10^{-10}$ with an effective measurement time of 8.63 *μ*s. Given the applicability of transmission estimation to a number of sensing protocols and that the required measurements to saturate the QCRB are readily available, the results presented here are expected to enable quantum-enhanced sensors that can surpass the classical state-of-the-art, and promise to have significant impact to a number of fields.

## Figures and Tables

**Figure 4:**
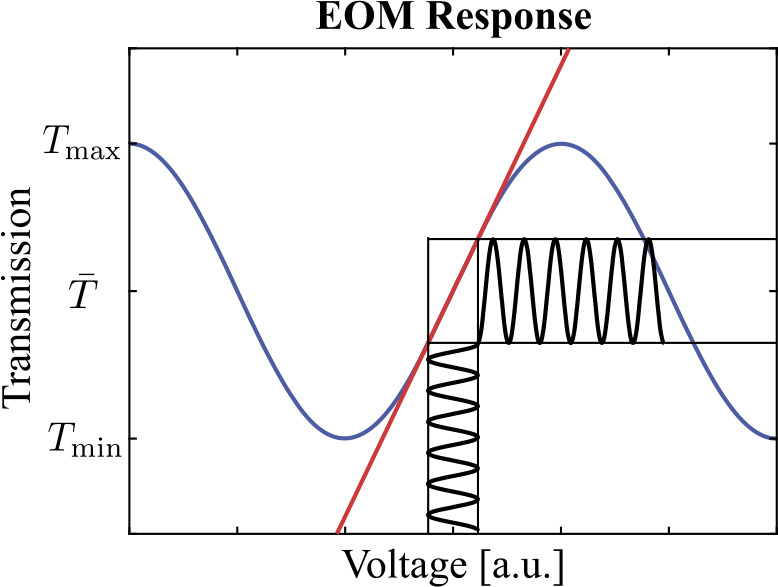
EOM setup response. The transmission through the EOM setup as a function of applied voltage across the EOM crystal is shown in blue. The red line marks the linear region of the EOM around the operating voltage used in the experiment, which is centered around *T̄*. A modulation of the voltage around this point, shown in black, results in a transmission modulation at the same frequency, as long as the modulation is within the linear regime.

**Figure 5:**
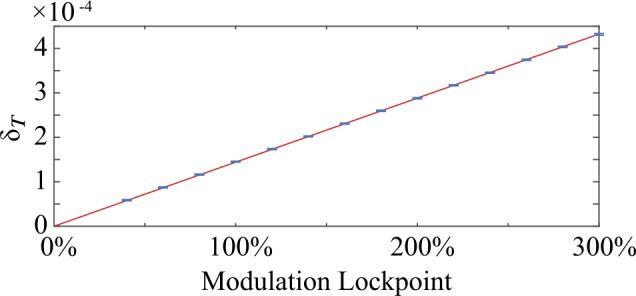
Calibration of the transmission modulation due to the EOM, $\delta _{T}$, as a function of its modulation lockpoint. The calibration data is shown in blue with one sigma error bars for the *y*-axis as the modulation lockpoint is scanned from 0% to 300%, with the scale set by the range of lockpoints used for the experiment. The red line gives the linear fit to the data and shows that even at three times the modulation depth used in the experiment the EOM response remains linear.

**Figure 6:**
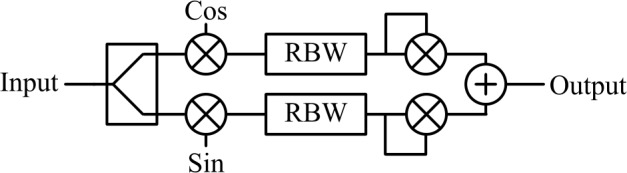
Schematic of a basic spectrum analyzer. For a given input *I*, the spectrum analyzer splits the signal into two channels and mixes each of the resulting signals with an electronic local oscillator (LO). The LOs used for the two channels are 90° out of phase to obtain the in phase and out of phase components. After mixing with the LO, the resulting signal in each channel is passed through a low pass filter, which represents the RBW filter of the spectrum analyzer, and then squared. Finally, the signals from both channels are summed to obtain the output. An additional filter, the video bandwidth filter, can be used on the output to reduce the fluctuations on the noise measurements. This filter, however, does not affect the mean value of the measured noise, and as a result it is not considered given that it does not play a role in our results.

## Data Availability

The datasets used and/or analysed during the current study are available from the corresponding author on reasonable request.

## Appendix

### A.1 Locking

To preform measurements that are able to saturate the QCRB, we need to stabilize multiple aspects of the experiment such as the number of photons used to probe the system under study and the transmission modulation amplitude. To keep the number of probing photons constant, the power of the probe beam after the FWM process needs to be stabilized. This requires keeping both the seed probe power and the gain of the FWM processes stable.

To stabilize the power of the seed probe for the FWM process, a portion of the probe beam is picked off via a half waveplate and PBS before the Rb vapor cell. This pick off is then detected with a photodiode and is used to obtain the error signal. The power of the seed probe is kept constant by controlling the diffraction efficiency of the AOM used to generate the probe from the pump beam. This setup is typically referred to as a noise-eater.

Given that the gain of the FWM process depends on the atomic number density, pump power, and frequencies of the involved fields, all of these have to be stabilized. The atomic number density depends on the temperature of the Rb vapor cell. Thus, a temperature controller is used to stabilize the Rb cell temperature to 120°C within a fraction of a degree. As with the probe beam, the pump power is locked before the Rb vapor cell by detecting a small portion of the pump that is picked off via a beam sampler. The pump power is then kept constant by feeding back to an electronically controlled rotation mount containing a half waveplate placed before a PBS. This feedback control is slow; however, the pump power changes are mostly due to slower thermal drifts and the intensity noise of the pump has little effect on the generated quantum state. Thus, a high bandwidth noise-eater like the one used for the probe, is not needed. Finally, since a change in the frequency of the laser changes the gain and thus the output conjugate and probe powers, we use the conjugate power to obtain the error signal to compensate for the frequency drifts. We use this approach as the probe transmission, and thus detected power, is changed as part of the experiment. The error signal obtained from the conjugate power is then fed back to lock the laser frequency, which results in a stable lock over the more than 20 hours needed to take the data.

The transmission modulation amplitude must also be controlled for reproducibility from data set to data set. This requires tight control of the transmission modulation amplitude ramp and control over the mean transmission through the EOM. In the implementation of the system, as shown in the “system” inset of Fig. 1, the EOM setup has a quarter waveplate and a half waveplate before the EOM and a half waveplate and a PBS after the EOM. The waveplates before the EOM, used for the amplitude modulation configuration, are set such that the EOM has a high transmission and the transmission modulation amplitude of the system, *δT*, is within the linear regime, as can be seen in Fig. 4. As such, the maximum, $T_{\max }$, and minimum, $T_{\min }$, transmissions through the EOM setup have to be properly set. For our experiment the ratio $T_{\max }/T_{\min }$ is set to around 1.02 with a mean transmission of 84%, giving a maximum change in transmission through the EOM of $\approx 0.8\%$ via the applied voltage. The maximum mean transmission through the system is limited by losses introduced by the various optical elements, in particular the EOM and the two PBSs shown in the “system” inset in Fig. 1. To lock the EOM, we detect the output reflection of the PBS of the amplitude modulation configuration. The output of the photodetector is split into DC and AC signals to lock the mean transmission and transmission modulation amplitude, respectively. The DC lock ensures the EOM operates in the linear regime by keeping the mean transmission at its center value, see Fig. 4. Locking the mean transmission at the center point, *T̄* in Fig. 4, provides the largest possible slope to maximize the transmission modulation amplitude for a given modulation voltage. Furthermore, if the mean transmission drifts, the calibration of the transmission modulation amplitude (see Appendix A.2) is no longer valid. The AC lock is used to control the transmission modulation amplitude during the amplitude ramp. The AC lock PID output is sent to the function generator used to create the modulation voltage sent to the EOM. The AC and DC voltage outputs from the locking electronics are combined with a Bias Tee. The combined signal is then amplified and sent to the EOM.

Another important aspect of being able to reach the QCRB is to filter out the classical technical noise in the optical state used to probe the system under study. While the power of the seed probe beam was stabilized with a noise eater, such a configuration is unable to reduce the intensity noise to the shot noise limit [55]. As a result, a cleanup cavity (Newport SuperCavity model SR-140-C) is used to reach the shot noise limit at our operating frequency of 1.5 MHz. We use the Pound-Drever-Hall [56, 57] locking technique to keep the cleanup cavity on resonance with the probe. An EOM is used to add a phase modulation to the probe mode before coupling it into the cleanup cavity. The modulation frequency is set to 10 MHz, which is significantly larger than the linewidth of the cavity (<0.6 MHz). As a result, the sidebands from the modulation are not transmitted by the cavity and the reflected light can be used to generate the required error signal.

### A.2 Calibrations

In order to compare the measured transmission uncertainties with the theoretical QCRB without any free parameters, the photon flux, propagation transmissions, and transmission modulation amplitude need to be properly calibrated.

To measure the propagation transmission we use two power meters. The first power meter, PM-A, is set on a flip mount in front of the Rb cell while the other, PM-B, is used to measure the transmission. First, we perform a relative calibration of the two power meters. We do this by placing PM-B right behind PM-A and measuring the power multiple times with both power meters by flipping PM-A in and out of the beam path. The ratio of the measured powers are then used to remove any systematic bias in the measurements. We then measure the transmission of the probe mode before the system under study without the Rb cell in place to be $T_{\text{common}}=98.4\%\pm 1\%$. This path has many common optical elements for the probe and conjugate mode paths. We then place the Rb cell back in place and, working off resonance, find the transmission for each of the cell windows to be $T_{\text{window}}=98.8\%\pm 1\%$. The quantum efficiency of the photodiodes are taken from previous calibration results to be $\eta =94.5\%\pm 2\%$ [41]. Altogether this leads to a probe transmission before the system of $T_{p}=T_{\text{common}}T_{\text{window}}=97.3\%\pm 1\%$, probe transmission after the system of $\eta _{p}=\eta =94.5\%\pm 2\%$, and a conjugate transmission of $\eta _{c}=T_{\text{common}}T_{\text{window}}\eta =91.9\%\pm 2\%$.

We operate the EOM in a regime in which the transmission modulation it introduces ($\delta _{T}$) is linear with the voltage applied across the EOM crystal. To calibrate the slope and thus the relation between $\delta _{T}$ and the applied voltage, we record the probe power oscillations on an oscilloscope for a given set of 14 modulation amplitude lockpoints. We then perform a fast Fourier transform of the recorded traces to isolate the amplitude of the oscillation from the noise and fit the transmission modulation to the applied voltage oscillation amplitude, as shown in Fig. 5. We consider a maximum modulation lockpoint of 300%, corresponding to a value three times higher than the maximum lockpoint value used in the experiment, due to the high electronic noise of the oscilloscope as compared to the spectrum analyzer. As can be seen in Fig. 5, the transmission modulation is linear with the applied voltage even at the higher lockpoints used for the calibration.

Finally, the transmission modulation amplitude due to the whole system under study, *δT*, for a given mean transmission, *T*, is given by $\delta T=T\delta _{T}$. As the mean transmission is reduced during each data set, the transmission modulation also decreases. This leads to the difference in scale between the *x*-axis of Fig. 2 and the *y*-axis of Fig. 5 in the range between 0% and 100% of the modulation lockpoint.

### A.3 Estimation of transmission uncertainty through modulation measurements

Given that we are interested in determining the uncertainty in the estimation of transmission, we now outline the procedure to obtain its value from the transmission modulation amplitude introduced by the system under study at a SNR = 1. To do this, we consider a small transmission modulation amplitude *δT* (defined as the standard deviation of the modulation) introduced by the system under study, which results in the modulation of the measured quantity *M* according to

14 $$ \delta M=\delta T \biggl\vert \frac{\partial M}{\partial T} \biggr\vert , $$

where *δM* is the blue trace in Fig. 2 and $\vert \partial M/\partial T \vert $ is the slope of that trace. Looking specifically at our measurement technique, we have that

15 $$\begin{aligned}& M = T \langle \hat{n}_{p} \rangle -g \langle \hat{n}_{c} \rangle , \end{aligned}$$

16 $$\begin{aligned}& \biggl\vert \frac{\partial M}{\partial T} \biggr\vert = \langle \hat{n}_{p} \rangle , \end{aligned}$$

where *g* in Eq. (15) is some electronic gain, and

17 $$ \bigl\langle \Delta ^{2}M \bigr\rangle =T^{2} \bigl\langle \Delta ^{2} \hat{n}_{p} \bigr\rangle +T(1-T) \langle \hat{n}_{p} \rangle - \frac{T^{2}\text{Cov}^{2}}{ \langle \Delta ^{2}\hat{n}_{c} \rangle } $$

for the optimal gain, with the term Cov given by the covariance between $\hat{n}_{p}$ and $\hat{n}_{c}$. For the bTMSS, it can be shown that

18 $$\begin{aligned} \frac{\delta M^{2}}{ \langle \Delta ^{2}M \rangle } =& \frac{\delta T^{2} \vert \alpha \vert ^{2}\cosh ^{2}(s)}{T^{2}\cosh (2s)+T(1-T)-T^{2} \left[\cfrac{\sinh ^{2}(2s)}{\cosh (2s)} \right]} \end{aligned}$$

19 $$\begin{aligned} =& \frac{\delta T^{2} \langle \hat{n}_{p} \rangle }{T-T^{2} [1-\operatorname{sech}(2s) ]} \end{aligned}$$

20 $$\begin{aligned} =&\frac{\delta T^{2}}{ \langle \Delta ^{2}T \rangle }, \end{aligned}$$

where the last equality is obtained by using the uncertainty in the estimation of transmission given by the QCRB for the bTMSS, $\langle \Delta ^{2}T \rangle $, as we have previously shown in [35]. Thus, the SNR of the measurement is equal to the SNR for transmission estimation.

It is important to note that such an equality is not specific to our measurement strategy and can be shown to be the case in general through error propagation; that is

21 $$ \text{SNR}=\frac{\delta M}{\Delta M}= \frac{\delta T \left \vert \frac{\partial M}{\partial T} \right \vert }{\Delta M}= \frac{\delta T}{\Delta T}. $$

Thus, when SNR = 1, the transmission modulation amplitude introduced by the system is equal to the standard deviation in the estimation of transmission, such that

22 $$ \Delta T=\delta T|_{\text{SNR}=1}. $$

We can further relate Δ*T* to the measured signal by combining Eqs. (22) and (21), such that when SNR = 1 we have that

23 $$ \Delta T=\Delta M \Big/ \biggl\vert \frac{\partial M}{\partial T} \biggr\vert , $$

with the uncertainty in the estimation of transmission then given by $\langle \Delta ^{2}T \rangle =(\Delta T)^{2}$. Such a relation between the SNR and the uncertainty in the estimation of a parameter at the QCRB has already been shown theoretically in [18, 58] and use in experiments to show the operation of an interferometer at the QCRB [59].

In our experimental procedure, we first calibrate the transmission modulation amplitude *δT* vs. the applied voltage to the EOM (see Appendix A.2). This then allows us to ramp down the calibrated transmission modulation amplitude *δT* to determine its value at a SNR = 1, marked with the circled “X” in Fig. 2. We finally take advantage of Eq. (22) to obtain the uncertainty in the estimation of transmission from the measurements for a given mean transmission *T* by taking the square of $\delta T|_{\text{SNR}=1}$.

### A.4 Photon counting

The dependence of the QCRB on the number of photons used to probe the systems under study makes it necessary to estimate such a quantity for the bTMSS, which is a continuous state. To go from a continuous photon flux to a discrete photon number requires bucketing the flux into discrete measurement time bins. The effective measurement time, *t*, for such a time bin is given by the response time of the measurement apparatus. In our experiments the measurement response is dominated by the spectrum analyzer, such that the effective measurement time is set by its RBW. To find the effective measurement time for a given RBW, we first find the relationship between the variance measured with the spectrum analyzer and the number variance of the detected optical state. We then take advantage of the known relationship between the number variance and mean photon number for the coherent state, that is $\langle \Delta ^{2}\hat{n} \rangle = \langle \hat{n} \rangle $.

We consider the basic spectrum analyzer design shown in Fig. 6 to determine *t*. Since the output, *O*, of the spectrum analyzer is proportional to the variance of the input, *I*, we first determine the proportionality constant, *K*, between these two quantities. In the experiment, the value of this constant has no effect on the number of photons measured, as it is purely a scaling factor due to the electronics and therefore needs to be taken into account. To find the proportionality constant, we first consider a deterministic signal $I_{\text{dtm}}=A\sin (2\pi ft+\phi )$ with variance $\langle \Delta ^{2}I_{\text{dtm}} \rangle =A^{2}/2$. If we set the frequency of the electronic local oscillator (LO) used to demodulate the in phase (cos) and out of phase (sin) components of the input to the same frequency as our deterministic signal, we get – after splitting, LO mixing, resolution bandwidth filtering, squaring, and summing (see Fig. 6) – an output of the form

24 $$ \langle O_{\text{dtm}} \rangle =\frac{A^{2}}{8} \bigl\vert H(0) \bigr\vert ^{2}=K\frac{A^{2}}{2}, $$

where $H(f)$ is the frequency response of the RBW filter. As a result,

25 $$ K=\frac{1}{4} \bigl\vert H(0) \bigr\vert ^{2}, $$

such that the ratio $\langle O \rangle /K$ gives the actual variance of the input, $\langle \Delta ^{2}I \rangle $. The number variance for an input state can then be related to the output of the spectrum analyzer through error propagation according to

26 $$ \bigl\langle \Delta ^{2}\hat{n} \bigr\rangle = \bigl\langle \Delta ^{2}I \bigr\rangle {\Big/} \biggl\vert \frac{\partial \langle I \rangle }{\partial \langle \hat{n} \rangle } \biggr\vert ^{2} =\frac{ \langle O \rangle }{K} {\Big/} \biggl\vert \frac{\partial \langle I \rangle }{\partial \langle \hat{n} \rangle } \biggr\vert ^{2}. $$

Next, we consider an input state given by a coherent state to take advantage of the relation between the number variance and the mean number of photons to calculate the effective integration time *t*. To do so we need to calculate the different terms on the right-hand-side of Eq. (26). The mean value for an input coherent state is given by

27 $$ \langle I_{\text{coh}} \rangle =C_{p\rightarrow i} \vert \alpha \vert ^{2}=C_{p \rightarrow i}\frac{ \langle \hat{n} \rangle }{t}, $$

where $C_{p\rightarrow i}$ is the gain of the photodetector, $|\alpha |^{2}$ is the mean photon flux of the coherent state, and $\langle \hat{n} \rangle $ is the average number of photons detected over measurement time *t*. The mean value of the output of the spectrum analyzer in this case corresponds to the noise power of the coherent state. The expectation value of the output can be shown to be given by

28 $$ \langle O_{\text{coh}} \rangle = \frac{ \vert \alpha \vert ^{2}C_{p\rightarrow i}^{2}}{2} \int _{-\infty}^{\infty } \bigl\vert H(f) \bigr\vert ^{2}\,df. $$

As a result, the variance of the input takes the form

29 $$ \bigl\langle \Delta ^{2}I_{\text{coh}} \bigr\rangle = \frac{ \langle O_{\text{coh}} \rangle }{K}=2 \vert \alpha \vert ^{2}C_{p \rightarrow i}^{2} \frac{\int _{-\infty}^{\infty } \vert H(f) \vert ^{2}\,df}{ \vert H(0) \vert ^{2}}. $$

We can see that Eq. (28) is proportional to $\int _{-\infty}^{\infty } \vert H(f) \vert ^{2}\,df$, while the output from a deterministic modulation input, Eq. (24), is proportional to $\vert H(0) \vert ^{2}$. This difference comes from the deterministic signal being a single frequency peak, such that the contribution to the power spectrum (output of spectrum analyzer) is dominated by the power in the deterministic signal. On the other hand, the intensity noise of the coherent state is broadband, such that the power is distributed over all frequency components of the system response.

To find the measurement time, *t*, we can use Eqs. (27) and (29) in Eq. (26) to find the number variance for a coherent state

30 $$\begin{aligned} \bigl\langle \Delta ^{2}\hat{n} \bigr\rangle _{\text{coh}} =&2 \langle \hat{n} \rangle t \frac{\int _{-\infty}^{\infty } \vert H(f) \vert ^{2}\,df}{ \vert H(0) \vert ^{2}} \end{aligned}$$

31 $$\begin{aligned} =& \langle \hat{n} \rangle , \end{aligned}$$

where the last line is a property of the coherent state. We can now set the right-hand-side of Eq. (30) equal to Eq. (31) to show that the effective measurement time is given by

32 $$ t= \frac{ \vert H(0) \vert ^{2}}{2\int _{-\infty}^{\infty } \vert H(f) \vert ^{2}\,df }. $$

For an ideal spectrum analyzer, the RBW filter has a Gaussian profile with the full-width at half-maximum given by the value of the RBW, such that

33 $$ \frac{ \vert H_{\text{gaus}}(f) \vert ^{2}}{ \vert H_{\text{gaus}}(0) \vert ^{2}}=\exp\left(- \frac{4\ln (2)\:f^{2}}{\text{RBW}^{2}}\right). $$

Therefore, the time for our measurements would ideally be

34 $$ t_{\text{gaus}}=\sqrt{\frac{\ln (2)}{\pi}}\frac{1}{\text{RBW}}\approx \frac{0.47}{\text{RBW}}. $$

However, real spectrum analyzers are only able to approximate a Gaussian filter. For our spectrum analyzer (Agilent model E4445A) the filter is a 4-pole synchronously tuned filter with a correction factor of ≈ 0.94 [60]. Thus, the actual effective measurement time for our system is given by $t\approx 0.44/\text{RBW}$.

Finally, to obtain the number of photons used in the experiment, we need to calibrate the photon flux, $\Phi = \langle \hat{n} \rangle /t$, for the probe power used in the experiment. The DC voltage output, $V_{\text{dc}}$, of the photodetectors used to detect the probe and conjugate modes are linearly dependent on the optical power, *P*, detected, such that $V_{\text{dc}}=mP$ with *m* giving the proportionality constant. We first calibrate *m* by flipped a power meter in and out of the beam path in front of the photodetector and measuring the optical power *P* and output voltage $V_{\text{dc}}$ for incident beams with different powers. This allows us to perform a linear fit to get an accurate measure of *m*. Thus, we can find the photon flux according to $P=\Phi hc/\lambda $, where *λ* is the wavelength of the probe mode (795 nm for our experiment), *h* is Planck’s constant, and *c* is the speed of light. The photon number is then given by

35 $$ \langle \hat{n} \rangle =\Phi t=\frac{\lambda}{hc} \frac{t}{m}V_{\text{dc}}, $$

where $V_{\text{dc}}$ is recorded for each transmission for each data set measured to determine the mean transmission *T* independently for each data point taken. The number of probing photons is calculated by bypassing the system under study and is also measured for each data point taken.

### A.5 Inferring the squeezing parameters

The effective squeezing parameter, *s*, and the transmission of the probe through the Rb cell, $T_{a}$, must both be estimated to find the state generated. The probe transmission measured without the pump field is not an accurate estimation of $T_{a}$ as the strong pump field leads to optical pumping, which modifies the transmission of the probe. To estimate *s* and $T_{a}$ we measure the noise properties of the generated bTMSS and compare the measured values with the theoretically calculated noise properties that take into account the distributed losses in the atomic medium.

For the generated bTMSS, we measure the balanced intensity-difference noise and the individual intensity noises of the probe and conjugate modes at 1.5 MHz, the operating frequency of the experiment, and subtract the electronic noise. These noises are then normalized by the corresponding shot noise and backtracked to obtain the normalized noises generated by the FWM process. We backtrack the noises by removing the effects of the loss from the Rb cell output window, optical path to the detectors, and the quantum efficiency of the detectors. This is done by using the relation

36 $$ \text{N}_{0}=\frac{\text{N}_{\text{m}}-(1-\eta )}{\eta}, $$

where N_0_ is the normalized noise directly generated by the source, N$_{\text{m}}$ is the measured normalized noise, and *η* is the total transmission, which is given by $T_{\text{p}}\eta _{\text{p}}$ for the probe beam and $\eta _{\text{c}}$ for the conjugate beam. As described above, both transmission are the same so we can take $\eta =\eta _{\text{c}}=T_{\text{p}}\eta _{\text{p}}$ to backtrack the intensity-difference noise.

To calculate the theoretically expected noise properties that take into account distributed losses in the source, we model the FWM in the atomic system as an infinite series of infinitesimal layers of two-mode squeezers and beam splitters (to take into account distributed losses) [41, 53]. Given that the frequency of the conjugate beam is far away from any atomic resonance, we assume that it does not experience any losses. Thus, $T_{a}$ represents the loss due to atomic absorption for the probe beam. The sum of all the infinitesimal squeezing parameters gives the effective value of *s* and the product of all the infinitesimal transmissions of the beam splitters gives $T_{a}$. For the theoretical normalized noises, we use the results given in [53] and verify them through the numerical approach given in [41]. The analytical solutions for the theoretical model for the normalized noises are given by

37 $$\begin{aligned}& \frac{ \langle \Delta ^{2} (\hat{n}_{p} - \hat{n}_{c} ) \rangle }{ \langle \hat{n}_{p} \rangle + \langle \hat{n}_{c} \rangle } = 1- \frac{2s\sinh ^{2} \left(\frac{\xi}{4} \right)}{\xi \cosh \left(\frac{\xi}{2}+\zeta \right)} -\sqrt{T_{a}} \frac{s\ln ^{2}(T_{a})\sinh ^{4} \left(\frac{\xi}{4} \right)}{2\xi ^{3}\cosh \left(\frac{\xi}{2}+\zeta \right)} , \end{aligned}$$

38 $$\begin{aligned}& \frac{ \langle \Delta ^{2}\hat{n}_{p} \rangle }{ \langle \hat{n}_{p} \rangle }= \frac{16s^{2} \left\{1-\sqrt{T_{a}} \left[1-\cos \left(\frac{\xi}{2} \right) \right] \right\}+\ln ^{2}(T_{a})}{\xi ^{2}} , \end{aligned}$$

39 $$\begin{aligned}& \frac{ \langle \Delta ^{2}\hat{n}_{c} \rangle }{ \langle \hat{n}_{c} \rangle } = \frac{16s^{2}\sqrt{T_{a}}}{\xi ^{2}}-1 - \frac{2\sqrt{T_{a}} \left[ (8s^{2}-\xi ^{2} )\cosh \left(\frac{\xi}{2} \right)+\xi \ln (T_{a})\sinh \left(\frac{\xi}{2} \right) \right]}{\xi ^{2}}, \end{aligned}$$

where $\xi =\sqrt{16s^{2}+\ln ^{2}(T_{a})}$ and $\tanh (\zeta )=\ln (T_{a})/\xi $.

We then determine *s* and $T_{a}$ by finding the values of these parameters that provided the best match between the theoretical model and measurements, in log scale, through the goodness-of-fit parameter $\chi ^{2}$ [61],

40 $$ \chi ^{2}=\sum_{i} \frac{ [\text{Measurement}_{i}-\text{Theory}_{i}(s,T_{a}) ] ^{2}}{\text{Variance of Measurement}_{i}}, $$

where the sum is over the three normalized noises. We minimize $\chi ^{2}$ using a differential evolution optimization algorithm [62, 63] to find $s=2.04$ and $T_{a}=71\%$ with a $\chi ^{2}=0.4563$. The uncertainty in the fitted parameters is found by varying their values until $\chi ^{2}$ increases by the reduced $\chi ^{2}$. The reduced $\chi ^{2}$ is $\chi ^{2}/\text{dof}$, where dof represents the degrees of freedom given by the number of measurements minus the number of parameters to fit. For our case the dof is 1, so $\chi ^{2}/\text{dof}=\chi ^{2}$. Thus, we determine the values of the parameters for which $\chi ^{2}$ is doubled to find the uncertainties for the parameters. Altogether, we find $s=2.04\pm 0.02$ and $T_{a}=71\%\pm 2\%$.

The optimization algorithm we used performs a differential evolution, which is a type of genetic algorithm. As such, it starts with a random set of possible solutions, tests how well each solution works using some goodness-of-fit parameter, and then mixes the solutions in an attempt to increase the goodness-of-fit. Here, minimizing $\chi ^{2}$ serves as our goodness-of-fit. To initialize the algorithm, we randomly created a population with 5000 points of *s* and $T_{a}$ values limited to $0\le s\le 3$ and $0.5\le T_{a}\le 1$. We then record the $\chi ^{2}$ value for each of these points.

After initializing the population, we mix the different elements in a way that optimizes towards the lowest $\chi ^{2}$ value. To do this we first find the optimal point, $P_{o}$, that has values of *s* and $T_{a}$ that give the lowest $\chi ^{2}$ of all the points in the population. We take this point to make new points for the next generation of population. We select one of the remaining 4999 points to possibly replace, $P_{i}$. To create the possible replacement point, $P_{r}$, we randomly select two more points from the current population, $P_{j}$ and $P_{k}$, such that $P_{o}\ne P_{i}\ne P_{j}\ne P_{k}$. We then create a vector pointing from $P_{j}$ to $P_{k}$ normalized by the limits placed on the parameters, that is, normalized by $\sqrt{(0-3)^{2}+(0.5-1)^{2}}$ for our case. We create the replacement point $P_{r}$ by adding this vector to $P_{o}$. If the new point is outside of the limits set for *s* and $T_{a}$ for one or both of the parameter values, the point is set to the closest limit. We then find $\chi ^{2}$ for $P_{r}$ and, if it is lower than the $\chi ^{2}$ of point $P_{i}$, we replace $P_{i}$ with $P_{r}$. Finally, we repeat this for all points that are not $P_{o}$, randomly picking new $P_{j}$ and $P_{k}$ for each one. To increase the algorithm’s ability to find global minimums in the presence of local minimums, we perform the replacement for only 70% of the time when $P_{r}$ is better than $P_{i}$.

There is no set limit to the number of iterations needed to find the minimum $\chi ^{2}$, so we iterate the algorithm until the spread of the population is orders of magnitude less than the uncertainty in the parameter values. Saving the $\chi ^{2}$ value for each point during the differential evolution gives a look at the $\chi ^{2}$ dependence on *s* and $T_{a}$, especially around the global minimum. Thus, we can find a circular region around the minimum $\chi ^{2}$ where its value is doubled to find the uncertainty in the transmission and squeezing parameter.

## Abbreviations

LIGO: Laser Interferometer Gravitational-Wave Observatory
QCRB: Quantum Cramér-Rao bound
vTMSS: vacuum two-mode squeezed state
bTMSS: bright two-mode squeezed state
FWM: four-wave mixing
AOM: acousto-optic modulator
SNR: signal-to-noise ratio
EOM: electro-optic modulator
PBS: polarizing beam splitter
RBW: resolution bandwidth
